# Revealing Deactivation Pathways Hidden in Time-Resolved Photoelectron Spectra

**DOI:** 10.1038/srep35522

**Published:** 2016-10-20

**Authors:** Matthias Ruckenbauer, Sebastian Mai, Philipp Marquetand, Leticia González

**Affiliations:** 1 Institute of Theoretical Chemistry, Faculty of Chemistry, University of Vienna, Währinger Str. 17, 1090 Vienna, Austria

## Abstract

Time-resolved photoelectron spectroscopy is commonly employed with the intention to monitor electronic excited-state dynamics occurring in a neutral molecule. With the help of theory, we show that when excited-state processes occur on similar time scales the different relaxation pathways are completely obscured in the total photoionization signal recorded in the experiment. Using non-adiabatic molecular dynamics and Dyson norms, we calculate the photoionization signal of cytosine and disentangle the transient contributions originating from the different deactivation pathways of its tautomers. In the simulations, the total signal from the relevant keto and enol tautomers can be decomposed into contributions either from the neutral electronic state populations or from the distinct mechanistic pathways across the multiple potential surfaces. The lifetimes corresponding to these contributions cannot be extracted from the experiment, thereby illustrating that new experimental setups are necessary to unravel the intricate non-adiabatic pathways occurring in polyatomic molecules after irradiation by light.

Time-resolved photoelectron spectroscopy (TRPES) is considered a versatile experimental tool to probe molecular excited-state dynamics in polyatomic molecules^1^,^2^,^3^,^4^,^5^,^6^,^7^,^8^,^9^. The goal of such experiments is to monitor the molecular pathways of a wavepacket previously launched in a neutral electronic excited state by an ultrafast pump pulse. Information about the electronic configuration of the molecule and the region of the potential energy surface that is visited in time is encoded in the distribution of the kinetic energy of the detached electrons and their angle-resolved distributions as a function of time^10^. Examples exist, where femtosecond TRPES experiments have proved very useful to identify transient species in photodissociation problems^3^ or to map rotational^11^ or vibrational wavepacket motion^12^,^13^,^14^,^15^,^16^,^17^,^18^. Lately, also time- and angle-dependent ion yields of molecular ion fragments created by strong-field ionization have been employed to disentangle different pathways in relaxation processes^19^. Despite TRPES being a well-established experimental method, the interpretation of non-adiabatic processes in polyatomic molecules is challenging because transitions between many different electronic excited states take place on an ultrafast time scale and numerous electronic and vibrational changes occur simultaneously^20^,^21^.

Ab initio calculations as well as non-adiabatic dynamics simulations can help in the endeavour of identifying the states involved in the ionization and following the vibrational dynamics on the states of interest. In many cases, however, only the neutral excited-state dynamics is simulated and then compared to pump-probe photoelectron yields, which reflect the neutral dynamics only indirectly. This simplified approach also relies on the assumption that high-lying states give a full ionization signal while no signal is obtained if the population has proceeded to low-lying states^22^. While it might be a sufficiently good approximation in simple cases, this procedure often gives only a rough first estimate of the signal measured experimentally. As a step further, pump-probe spectra have been computed using time-dependent perturbation theory (see e.g. ref. 23), discretized ionic continua (see e.g. ref. 24) or approximated neutral-to-ionic zero electron kinetic energy dipole couplings (see e.g. ref. 25). More involved approaches of computing directly time-resolved ionization probabilities and photoelectron angular distributions are quite challenging and are only starting to emerge^20^,^26^,^27^,^28^,^29^,^30^.

In this paper we report simulations of static and time-resolved photoelectron spectra of cytosine in its ground and excited states. The interpretation of cytosine photoelectron spectra and TRPES is particularly challenging since different tautomers are present in the sample. Here, we disentangle the individual lifetimes of the many deactivation pathways, which otherwise cannot be separated from the experimental^31^ photoelectron ionization yield. We show that the decomposition of the time-resolved signal into the different channels of the relevant tautomers is hardly possible without theoretical insight, here obtained from non-adiabatic molecular dynamics simulations.

The relaxation dynamics of cytosine after UV irradiation has been the subject of extensive time-resolved experiments in the past^31^,^32^,^33^,^34^,^35^,^36^,^37^. Kosma *et al.*^34^ and Ho *et al.*^35^ showed the dependence of the observed signals on the excitation energy of the pump laser used with the aim to partially discriminate contributions from keto- and enol-cytosine. More recently, Kotur *et al.*^36^ combined time- and mass-resolved photoionization with ab initio electronic structure theory in order to track the time constants of some selected excited-state dynamics pathways of the neutral molecule. While these experiments use strong-field multiphoton ionization as a probe, Ullrich *et al.*^31^ employed a 200 nm probe laser, which is capable of ionizing cytosine with a single photon from the excited states. Hudock and Martínez^28^ simulated the TRPES of keto-cytosine in the gas phase using Dyson norms and ab-initio multiple spawning dynamics on the singlet electronic excited states. In gas phase, however, cytosine shows a large contribution of the enol form^38^. Moreover, ongoing work in our group^39^,^40^,^41^ has shown that triplet states also participate to the ultrafast excited-state dynamics of cytosine. For an overview of additional literature on cytosine dynamics, the reader is referred to ref. 41.

In the present work, we use ab initio electronic structure calculations to obtain the energies and electronic configurations of the involved neutral and ionic states. Furthermore, we employ Dyson norms and non-adiabatic surface hopping trajectory methods to perform time-resolved photoelectron spectrum simulations of the relevant tautomers of cytosine. Most importantly, we show that the observed photoelectron signal does not reflect the underlying processes from the dynamics of the neutral molecules, pointing to a very complex excited-state dynamics which cannot be disentangled alone by single-photon ionization experiments.

## Methodology

### Theory

Ionization involves a transition (called ionization channel *i* → *α* here) from an *n*-electron source state *i*, with the wavefunction 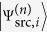, to an (*n* − 1)-electron ionic state *α*, with the wavefunction 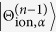 with associated orthogonal continuum function of the free electron 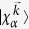. The latter is here assumed to be described in momentum space with the momentum vector 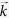. Assuming the sudden approximation^42^,^43^,^44^, the transition probability from a source state to an ionic state in dipole representation can be written as:


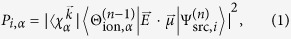


where 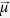 is the transition dipole moment operator and 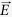 the electric field. Rigorous computation of *P*_*i*,*α*_, as denoted in Eq. (1), requires a treatment of the continuum wavefunction for the free electron, 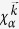, and the transition dipole moment between the molecular states^30^,^45^,^46^,^47^,^48^,^49^. These computations are very demanding, especially if many ionization probabilities along trajectories from molecular dynamics simulations need to be calculated. Going beyond the sudden approximation is possible but even more tedious^29^,^50^. Instead, a computationally more efficient procedure can be obtained by approximating the transition dipole moment as a constant and assuming 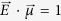, thereby reducing Eq. (1) to


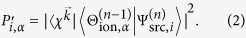


In this expression appears a quantity called *Dyson orbital*, which is defined as rescaled *ionization source orbital*:


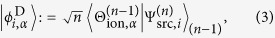


where the subscript to the braket indicates integration over the coordinates of (*n* − 1) electrons^27^,^29^,^50^. The norm of Dyson orbitals, called Dyson norm, can be used to obtain an adequate, efficient estimate of the photoionization yield for single photon ionization in the XUV regime but does not allow to calculate multiphoton (IR-, strong-field) ionization yields^29^.

Configuration interaction (CI) type wavefunctions, one of the most common wavefunction types in ab initio quantum chemistry, are a linear combination of Slater determinants


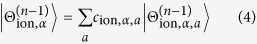



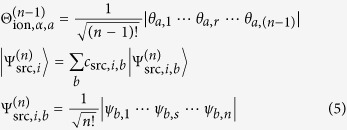


with orthonormal one-electron spin-space molecular orbitals *θ*_*a*,*r*_ and *ψ*_*b*,*s*_. The Dyson orbital for a pair of states *i* and *α* can thus be calculated as the sum of the overlaps of all Slater determinant pairs (indices *a*, *b*) weighted with the product of their CI coefficients:


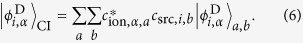


The contribution 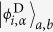 of one Slater determinant pair can be rewritten in terms of the molecular spin-orbitals of the source wavefunction, 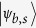, and the annihilation operator, 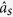, for the *s*-th occupied source spin-space orbital, 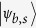:


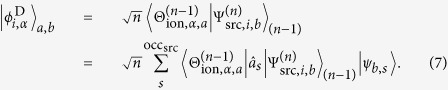


In order to obtain an expression for the ionization yield, Equation (2) can be rewritten in terms of the Dyson orbitals (dropping the constant 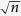 factor in Eq. (3))


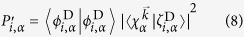


where 
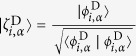
 is the normalized Dyson orbital. Note that the above equation is normalized to *δ*(*ħω* + *V*_*i*_ − *V*_*α*_ − *E*_*kin*_) with the frequency of the ionizing laser *ω*, the potential *V*_*i*,*α*_ of the states *i* and *α* at the current geometry, and kinetic energy of the outgoing electron *E*_*kin*_. Analogously to the definition of the oscillator strength for absorption spectroscopy, we define (integrating over all 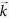-vectors) the amplitude *W*_*i*,*α*_ as the measure for the total yield of the ionization channel *i* → *α*:


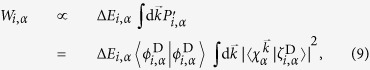


where Δ*E*_*i*,*α*_ is the energy difference between states *i* and *α* (the binding energy). This factor is used according to the general formulation of the oscillator strength. For a transition from a state *i* to a state *u*, the oscillator strength is 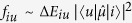 with the energy difference Δ*E*_*iu*_ between the states and the dipole operator 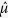 (assumed 1 in our case)^51^. Note that this formula also applies for ionization and the sum of all possible oscillator strengths originating from one state is equal to the number of excitable electrons *z* according to the *f*-sum rule: 
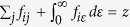
, where *ij* indicate all possible transitions from state *i* within the neutral molecule and *iε* stands for transitions to the continuum^51^. Under the assumption that the structure of the continuum-wavefunction of the ejected electron does not strongly depend on the remaining molecular wavefunction (which also implies that other factors, like the density of states, are constant):


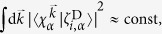


the amplitude *W*_*i*,*α*_, which we use as a measure for the ionization probability in our study, reduces to the Dyson norm times the binding energy of the corresponding ionization channel:


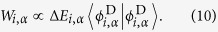


In this approach, the different ionization channels are uncoupled, which would basically amount to the first Born approximation, if the kinetic energy of the electrons was actually evaluated. Here, the approximation goes further because the Dyson norms are considered alone, which is equivalent to replacing the intensity at a particular photon energy by the integrated intensity over all photon energies and might be problematic for low kinetic energies of the outgoing electrons. Nevertheless, it is at least as good as a simple comparison of photon energy and ionization potential, and we choose it in the present study due to its high computational efficiency and since it can deliver reasonable or even correct results despite the many approximations, as shown e.g. in refs 20, 26, 52, 53, 54. It should be stressed that the focus of the present work is not to present accurate ionization calculations but we merely will show an example questioning the often assumed direct connection between the time dependence of the ionization signal and the dynamics of the neutral molecule.

### Computational Details

Dyson norms were calculated using multiconfiguration self-consistent field wavefunctions from Columbus^55^,^56^ with atomic integrals from Molcas^57^,^58^. In particular, singlet and triplet wavefunctions and energies were computed on the state-averaged complete-active-space self-consistent field (SA-CASSCF) level of theory with an active space of 12 electrons in 9 orbitals^40^ for all tautomers. Three singlet and four triplet states were averaged for enol-cytosine (abbreviated as (SA(3S + 4T)), while SA(4S + 3T) was used for keto-cytosine and SA(4S + 4T) for the imino tautomer. Doublet and quartet states were computed on the SA(6D + 6Q)-CASSCF(11,9) level of theory. The def2-svp basis set^59^ was applied throughout. The quality of the relevant ionized states was assessed with higher levels of theory, showing that CASSCF(11,9) describes the quartets and first four doublet states well, but performs poorly for higher excited doublet states. Since this makes the wavefunction and thus the Dyson norms for D_4_ and D_5_ unreliable, they are not included in the following discussion.

The CI-vectors, given in terms of configuration state functions (CSFs), were decomposed into Slater determinants since there is no analytic expression for the overlap of two CSFs. Only CI coefficients with a norm larger than 10^−4^ were included. Furthermore, the computation of Dyson orbitals included only determinant pairs with a product of CI coefficients larger than 10^−4^. A single-point test using the full CI vector and products of CI coefficients down to the threshold 10^−7^ showed no significant differences in the Dyson norms (the maximum deviation being ≈0.5%). The use of different CASSCF calculations for the neutral and for the ionic states implies that also the orbitals from these (*n*)-electron and (*n* − 1)-electron states differ. The overlap is simply calculated by taking a sum over the overlaps between the orbitals of the (*n* − 1)-electron wave function and the annihilation operator applied to the different orbitals of the (*n*)-electron wave function (see Equation (7)) within our general overlap code described in ref. 60.

**Figure 1:**
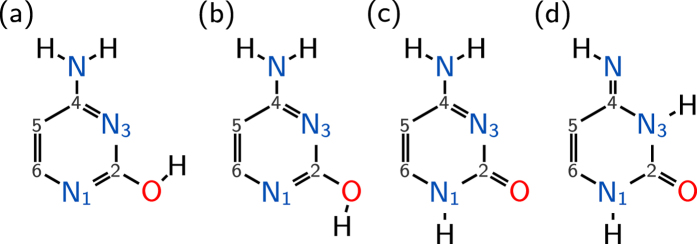
The investigated structures. (**a,b**) are the syn- and anti-rotamers of enol cytosine, (**c**) is keto-cytosine and (**d**) imino-cytosine. The terms syn and anti refer to the rotamers of the OH-group with respect to the -NH_2_ group. Hydrogens attached to carbons were omitted for clarity.

The photoelectron spectrum of cytosine was calculated using four tautomers: the syn- and anti-rotamers of the enol, the keto form, and the imino form, as shown in Fig. 1. The different tautomeric contributions were weighted as keto:syn:anti:imino = 29:17:37:17, in accordance to ref. 38. For each tautomer, 200 geometries were sampled randomly from a Wigner distribution generated using harmonic frequencies of the CASSCF(12,9) ground state minimum. At each sampled geometry, the Dyson norms for all applicable pairs of states were computed using the molecular orbital coefficients and CI-vectors from the quantum mechanical calculation. Based on the excitation energies and Dyson norms, a photoelectron spectrum was generated. Gaussian broadening was applied, using a full width at half maximum (FWHM) of 0.2 eV to smooth the spectra.

TRPES were calculated only for the keto and anti-enol tautomers, since the imino form has a higher first excitation energy than the other two and will therefore not be excited by the pump laser^31^. The anti-enol tautomer interconverts to the syn-enol one by a rotation about a single bond. This process occurs in 10% of the trajectories within 1 ps and since both types of structures are sampled, we abstain from separate calculations for the syn-enol tautomer. Dyson norms were evaluated every 5 fs on top of pre-computed neutral-dynamics trajectories^40^ obtained with the SHARC molecular dynamics suite^61^,^62^,^63^. Most of these trajectories were 1000 fs long. For a selection of 51 enol and 61 keto trajectories the simulation time was extended to 1400 fs. In SHARC, due to spin-orbit induced mixing, several states of different multiplicity can contribute at each instant of time to the active state^64^. Hence, the relative ionization probabilities to the different ionic states from the current neutral state in each single trajectory were determined as a sum of the Dyson norms of the according ionization channels, weighted with the contribution of the states to the trajectory’s wavefunction. As an isomer ratio we used keto:enol = 35:65, which corresponds roughly to the ratio in gas phase^38^. As the ground-state spectra, the simulated TRPES at each point in time were Gaussian-broadened with a FWHM of 0.2 eV. In order to compare the so-obtained spectra to the experimental results, we applied a Gaussian convolution in the time domain. The experimental data, which we compare to, contains a cross correlation with a width of 160 fs^31^, which was used as FWHM for the convolution.

## Results and Discussion

### Ground State and Excited State Photoelectron Spectra

**Figure 2:**
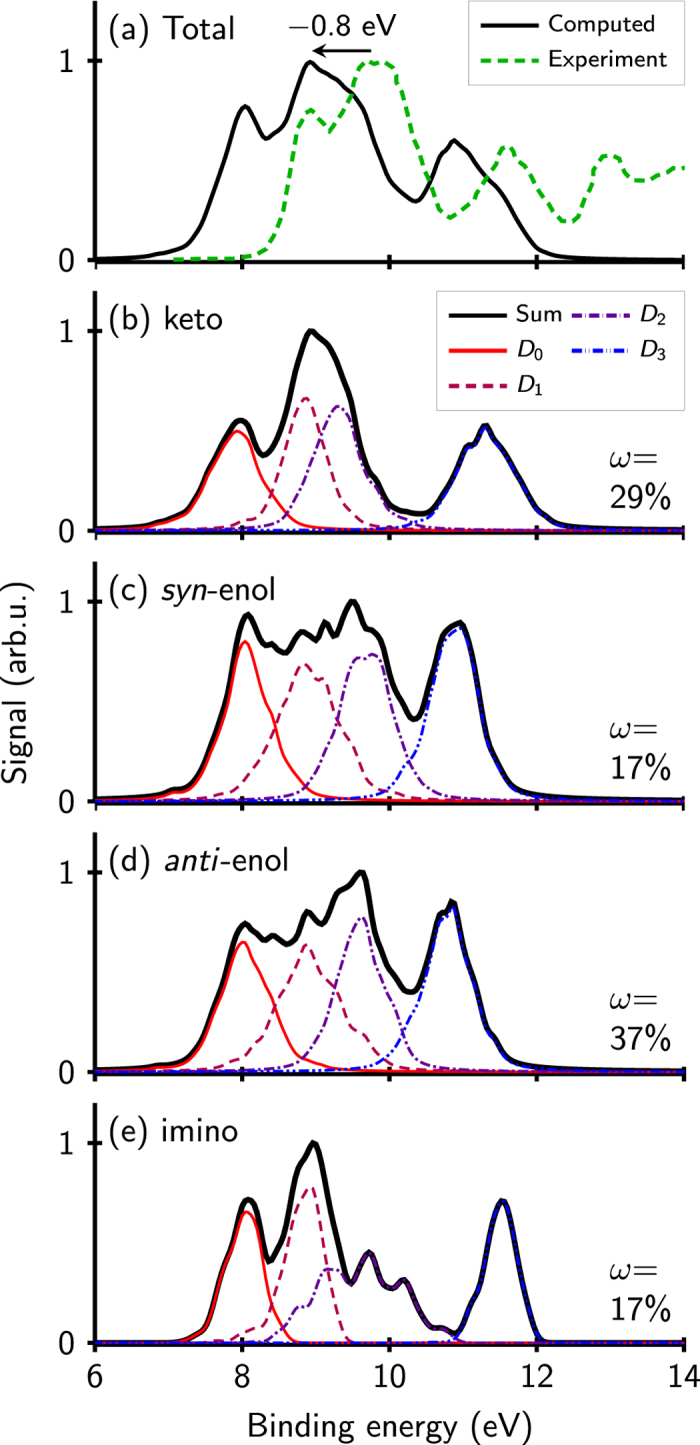
(**a**) Ground state computed photoelectron spectrum of cytosine in comparison to experimental data recorded at 80eV photon energy^65^. The energy bar displays the binding energy of the electron. Compared to the experiment, the calculated binding energy has a constant offset of ≈ −0.8eV. (**b–e**) Individual tautomer contributions to the photoelectron spectra, with their mixing weights *ω*. All spectra have been normalized to a maximum height of 1.0.

On the way to simulate the TRPES of cytosine, we computed first the ground state photoelectron spectra. All spectra presented in the following are reported in terms of the *binding energy*, i.e., the energy required to remove an electron completely and excite the remaining molecule to the respective doublet state. Figure 2(a) presents the simulated total ground-state spectrum of cytosine. The isomer-mixed S_0_-photoelectron spectrum is compared to experimental data recorded at 80 eV photon energy from Trofimov *et al.*^65^, which is reproduced also in other experimental studies, see e.g. refs 66 and 67. In the energy range from 6 eV to 12 eV, the spectral band shape is well reproduced. There exists a constant offset of ≈ −0.8 eV in the ionization potential, which is due to the lack of dynamical correlation in CASSCF^40^. This shift was taken into account in the computation of the TRPES (see below) by assuming a lower probe laser energy. The double peak at ≈10 eV in the experiment is only a shoulder in the peak at the respective energetic region (≈9 eV) of the calculated spectrum, indicating that the intensity of the D_2_-ionization is roughly 10% understimated. Otherwise the agreement between theory and experiment is excellent.

The individual ground state spectra of the tautomers are shown in Fig. 2(b–e), which also indicate the contributions of the ionization channels D_0_–D_3_. Table 1 shows the main electronic configurations of the neutral and ionic states. Using this information, it can be seen that the D_0_ to D_2_ peaks are due to ionizations from *π* orbitals located on the ring or nitrogen *n* orbitals. The only exception is the imino tautomer, where the D_2_ ionization channel arises from the oxygen *p*_O_ orbital, leading to a different D_2_ band shape in this tautomer. The D_3_ ionization channels differ between the isomers containing C=O and C–OH. In the former case, for keto- and imino-cytosine, the ionization occurs from an orbital containing the C=O *π* orbital, while in the enol forms the fourth excited doublet state has a hole in an *n* orbital. As a consequence, the D_3_ peak is for keto- and imino-cytosine well separated from the other peaks, whereas for enol-cytosine there exists a substantial overlap to the other peaks.

Photoionization spectra with excited states as source state have been also computed starting from the ground state equilibrium geometry. Particularly interesting is the spectra originated from the lowest-lying singlet states that are populated after the pump pulse, since this should be approximately equivalent to the TRPES for time delays close to zero.

At the present level of theory, keto-cytosine has—close to the ground state minimum structure—a crossing seam between S_1_ and S_2_. The states are strongly mixed which makes a uniform assignment of ππ^*^ and nπ^*^ difficult. Hence, it is advantageous to simulate the photoelectron spectrum not from either S_1_ or S_2_, but rather select for each geometry the ionization source states according to their approximated character, ππ^*^ or nπ^*^. This is done then for all the tautomers using the brightness of the S_0_ → S_*i*_ transition: the state with the largest transition dipole moment to S_0_ norm is assigned to ‘ππ^*^’ and the other one is assumed ‘nπ^*^’, even though we are aware that for strongly mixed states this assignment is not fully accurate. The S_3_ and S_4_ states of the keto- and imino-tautomers are neglected because they lie above the assumed excitation energy of 5.4 eV^40^.

The initial photoelectron spectrum will be a mixture of the two spectra with the ππ^*^ and nπ^*^ states as ionization source. The major contribution will come from the optically brighter state, although it is possible that some of the trajectories start in the optically less bright state, especially when the states are strongly mixed and the transition dipole moments are similar.

**Figure 3:**
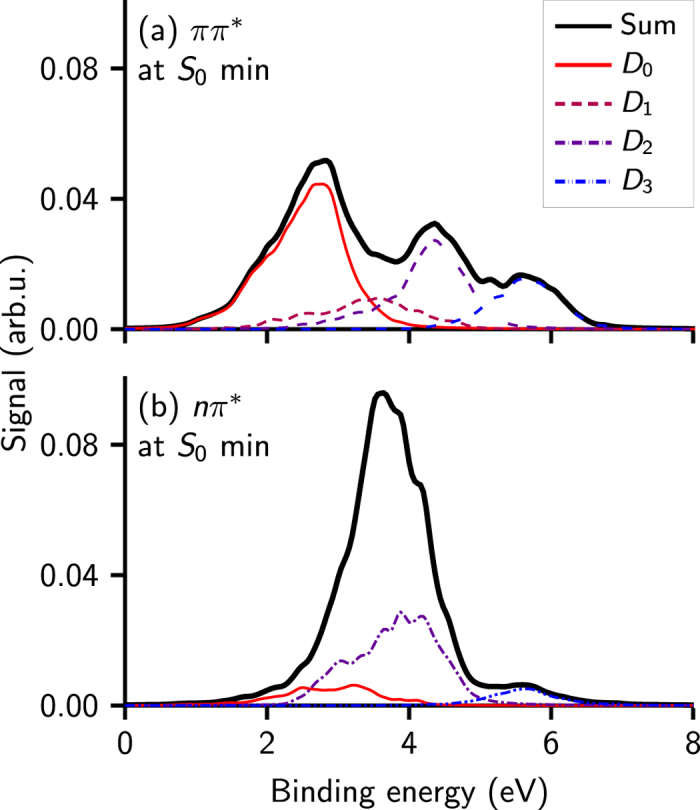
Photoelectron spectra of cytosine with (**a**) the bright (ππ^*^) and (**b**) the dark (nπ^*^) state as source states. Isomer-mixing weights as for S_0_ photoelectron spectra in Fig. 2. The curves have been scaled to reflect the intensities relative to the normalized S_0_ spectrum.

Figure 3 shows the computed spectra that stem from the ππ^*^ and nπ^*^ states, respectively. The ππ^*^-spectrum shows a strong band coming from D_0_ with some contributions of the D_2_ and D_3_ states while the nπ^*^-spectrum is dominated by the D_1_ and D_2_ peaks. This observation can be explained with the dominant wavefunction characters of the ionic states (see Table 1): Since for all tautomers the D_0_ is a *π* orbital-hole state (i.e., the singly occupied molecular orbital (SOMO) is a *π* orbital), the overlap with the ππ^*^ state is always large; thus, the D_0_ contributes strongly to the ππ^*^ spectrum in Fig. 3(a). In contrast, the overlap of a *π*-hole state with the nπ^*^ state is small. Therefore, the nπ^*^ spectrum shows no D_0_ contribution. The D_1_ contribution in the nπ^*^ spectrum originates from the enol and imino tautomers, because in these tautomers the D_1_ is an *n*-hole.

### Time-Resolved Photoelectron Spectra

**Figure 4:**
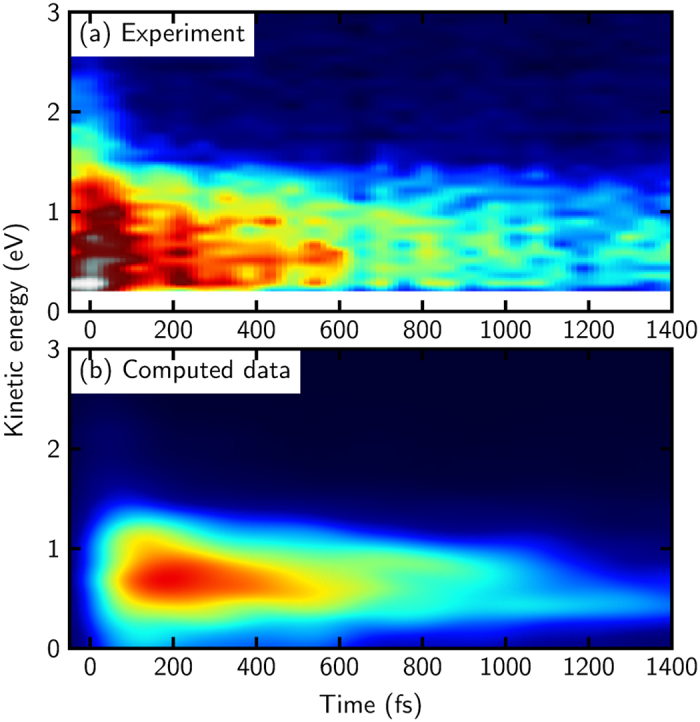
(**a**) Experimental^31^ and (**b**) computed time-resolved photoelectron spectra of cytosine. The coloring scheme has been adjusted to allow comparable visualization.

Figure 4 shows the experimental TRPES of Ullrich *et al.*^31^ and the simulated one. Experimentally, a 200 nm (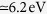) probe laser was employed so that it is possible to selectively ionize with a single photon from the excited states of cytosine without getting a signal contribution from the ground state. In order to account for the 0.8 eV downshift of the ionization potentials in our simulations (recall Fig. 2(a)), we assumed a lower probe laser energy of 5.4 eV.

**Figure 5:**
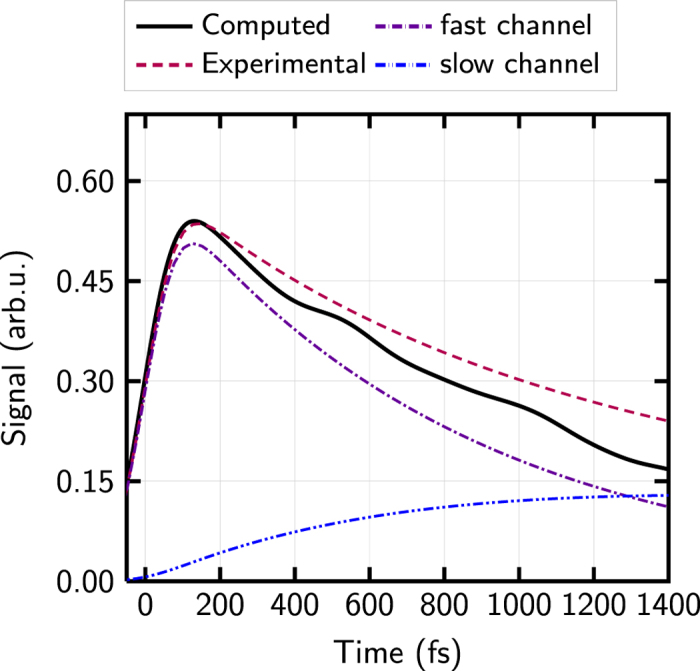
Energy-integrated (0 eV–4 eV kinetic energy) yield compared to experimental data from ref. 31. Experimental curves as dashed, computed as solid. The computed curve has been scaled to the same maximum value since it only gives relative intensities. The dot-dashed curves show the fast and slow contributions to the total pump-probe yield derived by Ullrich *et al.*^31^.

In the analysis reported by Ullrich *et al.*^31^, the energy-integrated experimental data was split into four contributions: (i) a probe-pump signal (when the probe-pulse precedes the pump-pulse), (ii) a Gaussian cross-correlation peak around 0 fs (where both laser pulses overlap), (iii) a fast decaying (‘short’) time pump-probe signal and (iv) a slowly decaying (‘long’) time pump-probe signal. Our calculations do not explicitly model the laser pulses; instead, the trajectories start at time *t* = 0 fs from a suitable distribution in the excited state (as is common practice in trajectory-based dynamics studies). Consequently, the first two of the contributions to the total signal, i.e., the probe-pump signal and the cross-correlation peak, are not included in the computations and indeed the area of high intensity around 0 fs and before 0 fs is missing in Fig. 4(b). The simulation can only reflect the pump-probe signals for *t* > 0 fs, which arise directly from the excited-state dynamics of the molecule. Having these facts in mind, the agreement of the computed data with the experimental is very good. If we extract the latter two contributions from the experimental data^31^ and compare their sum to the computed time-resolved (time-convolved) total yield integrated in the window of 0 eV to 4 eV kinetic electron energy, one sees that the agreement is excellent (see Fig. 5). The computed signal shows only a marginally faster decay than the experiment.

A detailed analysis of the contributions of the ionization channels to the total spectrum reveals that on average two to three, and in rare cases four, doublet states are accessed with the probe laser energy from the excited singlet and triplet states. No significant signal contribution from ionization to quartet states is observed in this energy range.

**Figure 6:**
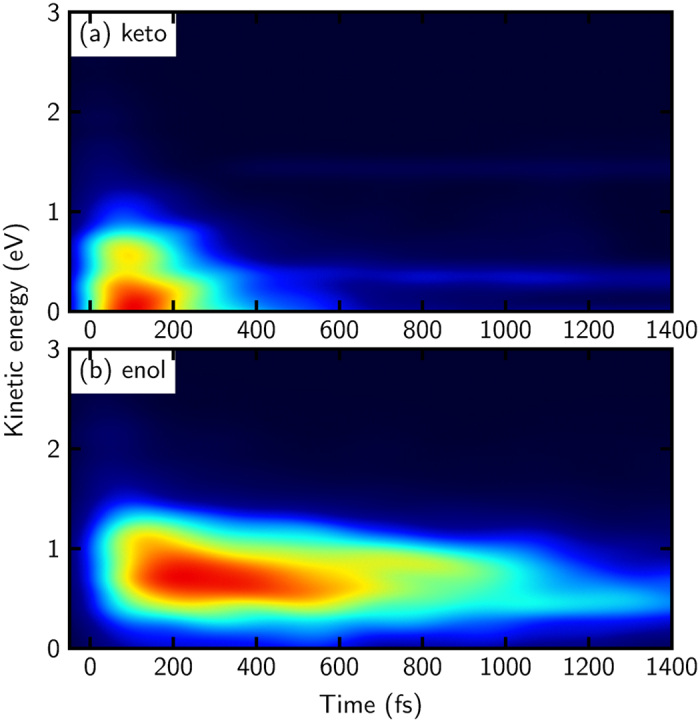
Computed individual TRPES of keto- (upper panel) and enol-cytosine (lower panel). For better visibility both signals are normalized.

The contributions of keto- and of enol-cytosine to the overall signal are plotted in Fig. 6. The keto contribution possesses a high peak at early times, which is situated at rather low kinetic energy. At later times, a small but almost constant tail is visible. The enol contribution is very similar to the total signal (Fig. 4(b)), which is not surprising, since enol-cytosine is the dominant tautomer in the gas phase. Notably, the enol spectrum decays much slower than the spectrum of the keto form.

**Figure 7:**
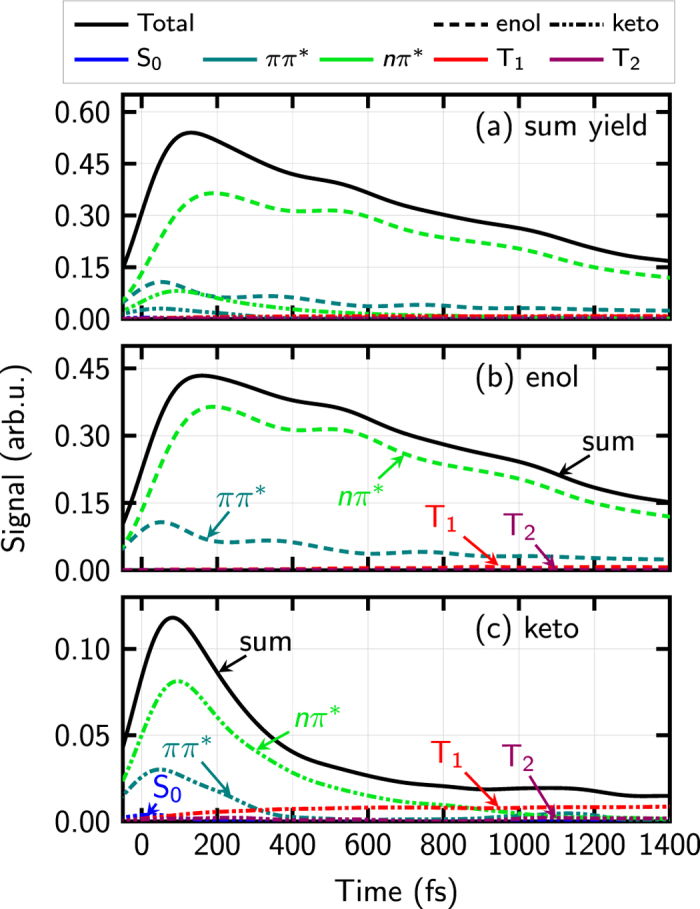
Computed photoelectron yield. The total sum (black curve in panel a) is the same as the black curve from Fig. 5. Additionally, the curve is decomposed into contributions from the different state populations *S*_0_, ππ^*^, nπ^*^, *T*_1_ and *T*_2_. For better visibility, the contributions are repeated separately for the enol (**b**) and keto (**c**) tautomers.

**Figure 8:**
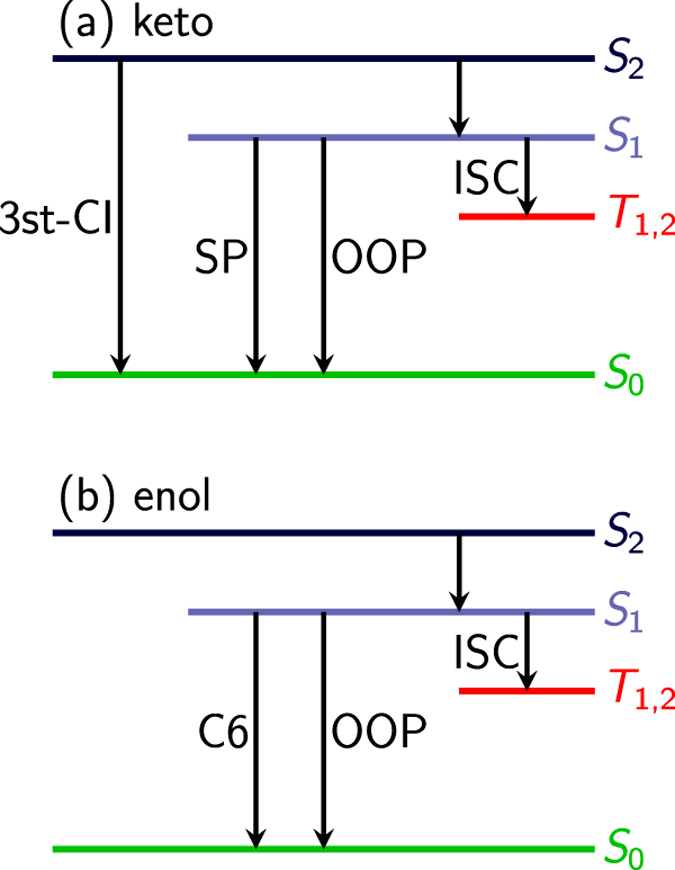
Schematic overview and naming scheme of the deactivation pathways for (**a**) keto- and (**b**) enol-cytosine as reported in ref. 40.

Figure 5 also shows the fit derived by Ullrich *et al.*^31^ of the total, energy-integrated experimental photoionization yield, to two time constants: a fast channel of 820 fs and a slow one of 3200 fs, where the latter contribution was taken from an earlier experimental study with multiphoton ionization^32^ and kept fixed in the fitting process. The intriguing question is now, which processes these two time constants can be attributed to. Since our simulations predict the same total photoionization yield, one could fit the exact same two time constants to our computed total signal, but such fitting would not provide any useful insight. Moreover, theory does not require such a fit because the population dynamics in the neutral molecules is directly available (unlike in the experiment, which targets to obtain neutral dynamics indirectly via the photoionization signal). Instead, we decompose the contributions of the different neutral state populations to the total photoionization signal. Such a decomposition is shown in Fig. 7 and as it can be seen, five components, and not two, are obtained. This is because at least five states (*S*_0_, *S*_1_, *S*_2_, *T*_1_ and *T*_2_, from which *S*_1_ and *S*_2_ are plotted as ππ^*^ and nπ^*^ states) are relevant in the relaxation dynamics of keto- and enol-cytosine. Figure 8 shows a summary of all the deactivation pathways that were found after excitation of keto- and enol-cytosine, according to ref. 40; as it can be seen a minimum of these five states are involved. Note that the ionization probabilities for the ππ^*^ and nπ^*^ states are approximately the same (see Fig. 3), any population excited initially to the ππ^*^ state quickly converts to the nπ^*^ state, and the enol tautomer is the most abundant one, leading to a dominant contribution of the enol nπ^*^ signal to the overall ionization yield.

A global fit of the time-evolution of the different Dyson norms provides the time scales reported in Table 2. Intriguingly, none of them agree with the two experimental values fitted by Ullrich *et al.*^31^. The main contribution, according to Fig. 7, is the decay of the enol nπ^*^ state and this state has a time constant of 1.1 ps, quite different from the experimental value of 0.82 ps or 3.2 ps. The latter values are rather a combination of different contributions –all hidden in the global photoionization signal.

Instead of fitting the photoelectron spectra, it is also possible to directly fit the population of the neutral nπ^*^ state. According to ref. 40, this yields a time constant of 2.4 ps. This value is also different from 1.1 ps, pointing to another problem that should be kept in mind when comparing theoretical neutral state dynamics with experimental photoelectron yields, or extracting neutral dynamics from them. The photoelectron signal is proportional to the time-dependent population multiplied with a time-dependent ionization probability. However, the kinetic models considered for fitting time constants rely on populations but exclude the time-dependent ionization probabilities, thereby preventing to extract the neutral state processes from the photoelectron signal, except in simple cases^20^,^21^. Only if the ionization probability from a distinct state is time-independent these simple kinetic models will suffice. Since, in general, the ionization probabilities change with geometry and electronic configuration and these will change during relaxation, the assumption of a time-independent ionization probability will not hold in many cases. To complicate it even more, different time constants may emerge for the same photoelectron signal if different kinetic models are assumed (even if only populations are considered instead of populations times ionization probabilities), making it extremely difficult to extract all the neutral excited-state pathways from photoelectron signals.

In order to obtain the neutral dynamics from an ionization experiment, different setups need to be devised. The approach of Kotur *et al.*^36^ is promising. Upon ionization, molecules can additionally break apart, leading to different molecular fragments. The analysis of not only the total ion signal but that of each molecular fragment, also with electronic structure calculations that helps identifying the fragments from which ionization takes place, provides neutral excited-state dynamics information. Other experimental setups can be conceived to decipher excited-state dynamics; in all of them, it is important to identify not only transitions between electronic states but the actual pathways taken, including multiple distinct pathways that could occur in parallel in a single electronic state.

**Figure 9:**
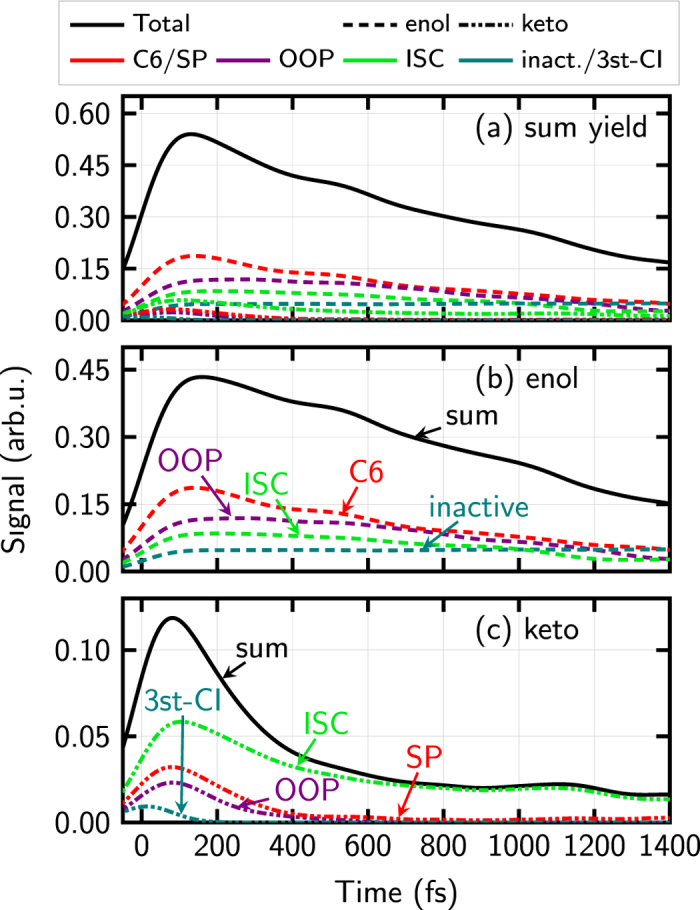
Computed photoelectron yield decomposed according to different pathways on the neutral potential energy surfaces, see text for details on the naming of the pathways. The total sum (panel a) is the same as the black curve in Fig. 5. The decomposition into the pathway contributions using information from our simulations show that it is impossible to do this decomposition from the total signal alone. For better visibility, the contributions from the enol tautomer (panel b) and keto tautomer (panel c) are shown separately.

Disentangling the multiple deactivation pathways is tedious but feasible with the help of dynamical simulations. A detailed analysis of our trajectories provides the following picture. The population predominantly starts in the bright (ππ^*^) state and then relaxes to the nπ^*^ state. Afterwards, the population either proceeds to the ground state (S_0_) or to a triplet state. For the latter two processes, four pathways have been identified in keto-cytosine (see Fig. 8): Ultrafast deactivation through a three-state conical intersection (‘3st-CI’), intersystem crossing (ISC), and two S_1_ → S_0_ deactivation pathways using two different conical intersections, one labelled out-of-plane (‘OOP’) and one semi-planar (‘SP’). The pathways are labelled according to the conical intersection leading to S_0_ or by ISC for intersystem crossing to the triplets. The enol-cytosine trajectories have been analyzed analogously, revealing three different pathways for excited-state deactivation, two using different conical intersections to the ground state (out-of-plane (‘OOP’) and C6-puckered (‘C6’)) and intersystem crossing to triplet states (‘ISC’). A small number of trajectories remained in the excited state throughout the full simulation time and are considered *inactive*. Table 3 collects the lifetimes of the different deactivation paths for the keto and enol tautomers, according to the above described conical intersections. The listed lifetimes have been fitted to independent monoexponential functions, since the respective pathways are considered independent. It is obvious that none of these estimated lifetimes or the corresponding contribution to the ionization signal, which is depicted in Fig. 9 can reasonably be extracted from the total isomer-mixed TRPES, without a priori theoretical insight. We therefore conclude with the present example that more elaborate experimental setups are needed to unravel the processes taking place during excited-state relaxation of molecules in general.

We argue that the above conclusions hold even if the ionization calculations are only approximate. A very good agreement with the experimental signal can be obtained by infinite combinations of relaxation pathways in the neutral molecule and geometry-dependent (and therefore time-dependent) ionization probabilities. Combinations stemming from processes with different durations may lead to averaged signals which cannot be disentangled afterwards. Our simulations represent one imaginable such combination being very different from the experimentally attributed time constants. Thus, they prove the afore-said by counter-example to the standard interpretation, i.e., where it is assumed that time constants from the ionization signal are directly related to the time constants of neutral-state processes.

## Conclusions

Cytosine is an excellent example to demonstrate that single-photon time-resolved photoionization alone is not sufficient to identify the hidden excited-state deactivation dynamics in the neutral molecule. Using Dyson orbitals in combination with non-adiabatic dynamics simulations we have reproduced the experimental pump-probe photoelectron spectrum available for cytosine^31^, including all relevant tautomers. An in-depth analysis of the simulated data reveals multiple processes contributing to the excited-state decay of the neutral molecules. The seven estimated time constants for these pathways are obscured in the experimental spectrum due to the additional time dependence of the conditions for ionization and cannot be recognized any more in the final simulated time-resolved photoelectron spectra without insight from dynamics simulations. The fitted experimental signals (3.2 ps and 820 fs) are a combination of multiple time constants stemming from a wealth of processes present in the different tautomers of cytosine in the gas phase. Experimental differentiation of the processes underlying the time constants requires extensive back-up by theory and more elaborated experimental setups.

## Additional Information

**How to cite this article**: Ruckenbauer, M. *et al.* Revealing deactivation pathways hidden in time-resolved photoelectron spectra. *Sci. Rep.* **6**, 35522; doi: 10.1038/srep35522 (2016).

## Figures and Tables

**Table 1:**
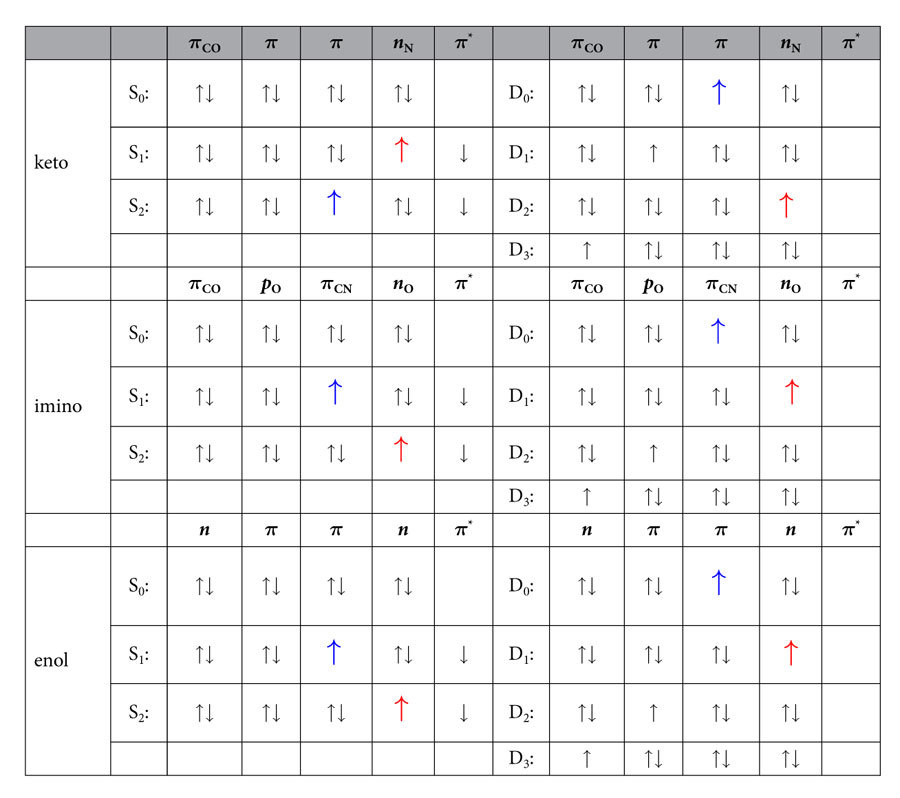
Main configurations of the relevant singlet and doublet states.

The contribution of the configuration to the respective state in percent is given in the respective last column. The first four doublet states are ‘single hole’ states, differing from the S_0_ configuration only by removal of one electron from a doubly occupied orbital. Subscripts to orbitals indicate the predominant location of the orbital, e.g. CO: C=O double bond, CN: C=N–H double bond. No subscript in the *π* orbital indicates *π*-orbital localized on the ring. For the sake of easier comparison between singlets and doublets, arrows corresponding to electrons in important singly occupied orbitals are colored.

**Table 2:** Fitted time constants for the integrated ionization yields depicted in Fig. 7.

Contribution	Time constant (fs)
— Enol —	
ππ^*^	57
nπ^*^	1080
— Keto —	
ππ^*^	21
ππ^*^	960
T_1_ + T_2_	310

For the respective global fit of each tautomer only the shown components have been considered.

**Table 3:** Fitted time constants for the integrated ionization yields depicted in Fig. 9 for the enol- and keto-cytosine.

Contribution	Time constant (fs)
— Enol —	
C6	886
OOP	1192
ISC	1277
— Keto —	
3st-CI	19
OOP	168
SP	184
ISC	316
